# Acetate-Inducing Metabolic States Enhance Polyhydroxyalkanoate Production in Marine Purple Non-sulfur Bacteria Under Aerobic Conditions

**DOI:** 10.3389/fbioe.2019.00118

**Published:** 2019-05-28

**Authors:** Mieko Higuchi-Takeuchi, Keiji Numata

**Affiliations:** Biomacromolecules Research Team, RIKEN Center for Sustainable Resource Science, Saitama, Japan

**Keywords:** polyhydroxyalkanoate, marine purple non-sulfur bacteria, aerobic conditions, TCA cycle, acetate

## Abstract

Polyhydroxyalkanoates (PHAs) are a family of biopolyesters that a variety of microorganisms accumulate as carbon and energy storage molecules under starvation conditions in the presence of excess carbon. Anoxygenic photosynthetic bacteria exhibit a variety of growth styles and high PHA production activity. Here, we characterized PHA production by four marine purple non-sulfur bacteria strains (*Rhodovulum sulfidophilum, Rhodovulum euryhalinum, Rhodovulum imhoffii*, and *Rhodovulum visakhapatnamense*) under different growth conditions. Unlike the well-studied PHA-producing bacteria, nutrient limitation is not appropriate for PHA production in marine purple non-sulfur bacteria. We found that marine purple non-sulfur bacteria did not accumulate PHA under aerobic conditions in the presence of malate and pyruvate. Interestingly, PHA accumulation was observed upon the addition of acetate under aerobic conditions but was not observed upon the addition of reductants, suggesting that an acetate-dependent pathway is involved in PHA accumulation. Gene expression analysis revealed that the expression of isocitrate dehydrogenase in the tricarboxylic acid (TCA) cycle decreased under aerobic conditions and increased with the addition of acetate, indicating that TCA cycle activity is involved in PHA production under aerobic conditions. We also found that expression of PdhR_rs_, which belongs to the GntR family of transcription regulators, in *Rhodovulum sulfidophilum* was upregulated upon the addition of acetate. Taken together, the results show that the changes in the metabolic state upon the addition of acetate, possibly regulated by PdhR, are important for PHA production under aerobic conditions in marine purple non-sulfur bacteria.

## Introduction

Polyhydroxyalkanoates (PHAs) are a family of biopolyesters synthesized by a wide variety of microorganisms as intracellular carbon and energy storage molecules (Lee, 1996a; Rehm, 2003; Nomura and Taguchi, 2007). Recently, PHAs have attracted attention as alternatives to petroleum-derived plastics because of their biodegradability and biomass origin (Choi and Lee, 1999; Martina and Hutmacher, 2007). Because PHAs are produced in the presence of excess carbon, the high production cost of carbon sources such as plant oils and sugars is one of the most important issues associated with PHA production. The use of photosynthetic organisms to produce materials is a potential method for reducing PHA production costs. Many studies have focused on higher plants and cyanobacteria as PHA producers that directly utilize carbon dioxide (CO_2_) (Mittendorf et al., 1998; Mooney, 2009; Osanai et al., 2013).

Anoxygenic photosynthetic bacteria, especially purple non-sulfur bacteria, are known to produce PHA at high levels (Liebergesell et al., 1991; Higuchi-Takeuchi et al., 2016) and have nitrogen fixation as well as CO_2_ fixation abilities (Tabita, 1995; Joshi and Tabita, 1996; McKinlay and Harwood, 2010). Purple non-sulfur bacteria perform three important biological reactions that sustain almost all organisms: light energy conversion, CO_2_ fixation and nitrogen fixation. Therefore, the utilization of purple non-sulfur bacteria is an environmentally friendly method for the production of useful materials. According to a study on the life cycle assessment (LCA) of PHA (Akiyama et al., 2003), we can reduce the cost of carbon and nitrogen sources for cultivation using their photosynthesis and nitrogen fixation abilities if we use marine purple non-sulfur bacteria as a host for PHA production. Purple non-sulfur bacteria exhibit a wide range of growth modes. A majority of purple non-sulfur bacteria can grow as photoautotrophs or photoheterotrophs in light, and some strains can grow aerobically in the dark as chemoheterotrophs. This variety of growth modes offers possibilities for a wide range of applications. Many application-based studies, such as studies on hydrogen production, vitamin production and bioremediation, have been carried out using anoxygenic photosynthetic bacteria (Sasikala and Ramana, 1995; Levin et al., 2004; Idi et al., 2015).

We focused on marine purple photosynthetic bacteria because high salinity can reduce the risk of biological contamination; and seawater, which is highly abundant and a low cost medium, can be used as a growth medium. Although PHA production by purple non-sulfur bacteria has been studied using freshwater strains of *Rhodospirillum rubrum* (Clemente et al., 2000) and *Rhodobacter sphaeroides* (Brandl et al., 1991), little is known about PHA production in marine purple photosynthetic bacteria. In our previous study, we demonstrated that marine purple non-sulfur bacteria were able to produce PHA under nutrient-rich growth conditions, unlike other well-known PHA-producing bacteria, which require nutrient-limited conditions (Higuchi-Takeuchi et al., 2016). We also revealed that the properties of the PHA synthase (PhaC_Rs_) from *Rhodovulum sulfidophilum*, a marine purple non-sulfur bacterium, are quite different from those of well-known PhaC proteins (Higuchi-Takeuchi et al., 2017). PhaC_Rs_ exists predominantly as an active dimer even in the absence of substrate, and PhaC_Rs_ activity increased with increasing substrate concentration.

In the present study, we examined PHA production by marine purple non-sulfur bacteria grown under different light and oxygen conditions. We found that PHA production was dependent on cell growth in the case of marine purple non-sulfur bacteria, unlike the PHA production observed in well-known PHA-producing bacteria. The marine purple non-sulfur bacteria did not accumulate PHA under aerobic conditions in the presence of malate and pyruvate, whereas PHA accumulation was greatly induced by the addition of acetate. Expression analysis suggested that tricarboxylic acid (TCA) cycle activity and expression of the GntR family of transcription regulators were involved in the enhancement of PHA production by the addition of acetate under aerobic conditions in *R*. *sulfidophilum*.

## Materials and Methods

### Culture Conditions

The marine purple non-sulfur bacteria investigated in this study were obtained from the biological resource centers (RIKEN BioResource Center, DSMZ, and ATCC) listed in Table S1. The marine purple non-sulfur bacteria were cultured in growth medium containing the following components per liter: KH_2_PO_4_, 0.5 g; CaCl_2_·2H_2_O, 0.25 g; MgSO_4_ ·7H_2_O, 3.0 g; NH_4_Cl, 0.68 g; NaCl, 20 g; sodium malate, 3.0 g; sodium pyruvate, 3.0 g; yeast extract, 0.4 g; ferric citrate, 0.25 mg; vitamin B12, 2 mg; ZnCl_2_·5H_2_O, 70 μg; MnCl_2_·4H_2_O, 100 μg; H_3_BO_3_, 60 μg; CoCl_2_·6H_2_O, 200 μg; CuCl_2_·2H_2_O, 20 μg; NiCl_2_·6H_2_O, 20 μg; and Na_2_MoO_4_·H_2_O, 40 μg. The pH was adjusted to 6.8 with 5 M of NaOH. The photosynthetic purple bacteria were grown under continuous far-red LED light conditions, as described below, at 30°C in screw-capped glass tubes, vials or plastic tubes.

### Nutrient-Limited Culture Conditions

For the nitrogen-limited conditions, NH_4_Cl, sodium malate and sodium pyruvate were removed from the growth medium, and 5 g of sodium acetate per liter was added as a carbon source. For phosphate-limited conditions, KH_2_PO_4_ was removed from the growth medium and supplemented with 0.28 g of KCl, and 5 g of sodium acetate per liter was added as a carbon source. The photosynthetic purple bacterial cells were cultured in growth medium and harvested during the log phase (OD_660_ = ~2.0) and then washed with nitrogen or phosphate-limited medium. The washed cells were diluted to a starting OD_660_ of 0.1 in nitrogen- or phosphate-limited medium. Screw-capped glass tubes were filled with medium to the tops of the necks and cultured at 30°C with agitation under 8 W/m^2^ of 730-nm LED light.

For vitamin-free conditions, vitamin B12, yeast extract, sodium malate and sodium pyruvate were removed from the growth medium, and 5 g of sodium acetate per liter was added as a carbon source. Cells were cultured in growth medium and harvested in the late log phase (OD_660_ = ~2.0–3.0). Harvested cells were washed with vitamin-free medium two times. For the two-stage culture method, cell cultures were diluted to a starting OD_660_ of 1.0 with and without vitamin mix (1 mg/L thiamine, 1 mg/L nicotinic acid, 0.6 mg/L 4-aminobenzoic acid, 0.1 mg/L biotin, 2 mg/L vitamin B_12_) and cultured at 30°C under 8 W/m^2^ of 730-nm LED light in screw-capped glass tubes filled with medium with agitation for 2 days.

### Growth Light Conditions

LED lights with wavelengths of 730 nm (VBP-L24-C3, Valore, Tokyo, Japan), 800 nm (ISC-201-2, CCS, Kyoto, Japan) and 850 nm (LFX2-200IR850, Marubishi Bioengineering, Tokyo, Japan) were used as light sources at the indicated intensities. Cells were cultured in growth medium and harvested during the log phase (OD_660_ = ~2.0). The harvested cells were diluted to a starting OD_660_ of 0.1 and cultured for 1 to 4 days at 30°C in a 15-ml plastic tube filled with medium to the top of the neck of the tube without agitation.

### Aerobic and Anaerobic Culture Conditions

The 25 ml glass vials were used under different oxygen concentrations in **Figure 3A**. Under aerobic conditions, 7 ml of log phase cells were cultured at 30°C in the dark using 25-ml vials with silicon plugs with shaking at 180 rpm. For oxygen-limited conditions, screw-capped vials were filled with medium to the tops of the necks. For anaerobic conditions, 10 ml of cell culture was added to 25-ml vials, and nitrogen gas was bubbled for 10 min. For all the conditions, the cells were diluted to a starting OD_660_ of 0.1 and cultured in growth medium at 30°C for 2 days.

For the aerobic cultures in **Figures 3B**, **4**, cells were cultured in the growth medium and harvested during the log phase (OD_660_ = ~2.0). Fifty milliliters of log phase cells were cultured at 30°C in the dark using 200-ml flasks with silicon plugs with shaking at 180 rpm. The harvested cells were diluted to a starting OD_660_ of 0.1 and cultured for 2 days. For PHA production under aerobic conditions in the presence of chemicals, cells were cultured with 10 mM dimethyl sulfide (DMS), 10 mM trimethylamine (TMA) and 10 mM thiosulfate, and 20 mM sodium bicarbonate, 0.5% sodium succinate and 0.5% sodium acetate.

### Analysis of PHA Content

The method used for PHA content characterization was modified slightly from a previous study (Chuah et al., 2013). The DCWs of lyophilized cells were determined gravimetrically. Approximately 0.5–2 mg of lyophilized cells was subjected to methanolysis in the presence of 1,000 μl of chloroform, 150 μl of sulfonic acid and 850 μl of methanol at 100°C for 140 min. After cooling, phosphate buffer (pH 8.1) was added to the reaction mixture, which was then neutralized with 5 N NaOH. After centrifugation at 1,500 rpm for 5 min, the bottom chloroform layer was dried over anhydrous sodium sulfate. The PHA content was determined using a gas chromatography-mass spectrometry (GC-MS) apparatus (GCMS-QP2010 Ultra, Shimadzu, Kyoto, Japan) equipped with a 30 m × 0.25 mm DB-1 capillary gas chromatography column (Agilent Technologies, CA, USA). For analysis, a 1-μl volume of sample solution was injected with helium as a carrier gas (3.30 ml min^−1^). The following temperature program was used to separate methyl esters: 45°C for 1 min, followed by a temperature ramp of 7°C per min to 117°C. The interface and ion source temperatures were 250°C and 230°C, respectively. The 3HB content was determined using a calibration curve.

### Quantitative Reverse Transcription Polymerase Chain Reaction (RT-PCR) Analysis

Under aerobic conditions, cells were cultured in growth medium and harvested during the log phase (OD_660_ = ~2.0). The harvested cells were diluted to a starting OD_660_ of 0.1 and cultured using 200-ml flasks with shaking in the dark for 2 days. Cells were also cultured under aerobic conditions in the presence of 5 g of sodium acetate per liter for 2 days. Under anaerobic conditions, log-phase cells were transferred to 15-ml plastic tubes and nitrogen gas was bubbled for 10 min and cultured under far-red light (30 W/m^2^: 730 nm) for 2 days. For different growth light intensity conditions, cells were cultured in 15-ml plastic tubes filled with medium to the top of the neck of the tube without agitation in low (8 W/m^2^: 800 nm) and high (50 W/m^2^: 800 nm) light for 3 days. All cells were frozen by liquid nitrogen and stored at −80°C until use.

Total RNA was extracted from *R. sulfidophilum* cells using the RNAeasy Mini Kit (Qiagen, Tokyo, Japan). Using 0.1 to 1.0 μg of RNA as a template, cDNA was synthesized by the QuantiTect Reverse Transcription Kit (Qiagen, Hilden, Germany) following the manufacturer's protocol. Quantitative reverse transcription PCR (RT-PCR) was performed using SsoAdvancedTM Universal SYBR Green Supermix (BIO-RAD, Hercules, CA, USA). The real-time RT-PCR analysis was performed by StepOne (ThermoFisher Scientific, Waltham, MA, USA) according to the manufacturer's protocol. The PCR conditions were composed of an initial denaturation step at 95°C for 10 min, 40 cycles at 95°C for 15 sec, and 60°C for 15 sec.

Melting curves were generated after amplification. The primer sets used to determine expression levels were designed by GENETYX Ver.13 and shown in Table S2. The specificities of primers were evaluated by melting curve analyses. Efficiencies were estimated from standard curves based on two-fold dilutions. The relative quantification in gene expression was determined using the standard curve method. Three to four biological replicates and four technical replicates of each biological replicate were used. The *rpoD* gene was used as a housekeeping gene to normalize the expression levels of target genes.

### Statistical Analysis

Statistically significant differences between groups were determined by the Student *t*-test, where a *p* < 0.05 indicated a significant difference.

## Results and Discussion

### Nutrient Limitation for PHA Production

PHA is known to be accumulated when excess carbon is present and other nutrients, such as nitrogen, phosphorus and sulfur, are limited (Valappil et al., 2008). In the case of marine purple non-sulfur bacteria, it was reported that PHA production was induced under nitrogen-limited (Liebergesell et al., 1991) or vitamin-limited conditions (Chowdhury et al., 1996). However, our previous study demonstrated that PHA production in some marine purple non-sulfur bacterial strains was not enhanced under nitrogen-limited conditions (Higuchi-Takeuchi et al., 2016). Time-course analysis of PHA contents (wt%) was performed using four marine purple non-sulfur bacterial strains (Table S1, *R. sulfidophilum, R. euryhalinum, R. imhoffii* and *R. visakhapatnamense*) under nitrogen-limited conditions and compared with under nutrient-rich conditions (Figure S1). PHA contents between 1 day and 5 days of cultivation were compared both under nutrient-rich and nitrogen-limited conditions in four strains. Significant differences were found under nutrient-rich conditions in all strains (*R. sulfidophilum*; *p* = 0.002, *R. euryhalinum*; *p* = 0.025, *R. imhoffii*; *p* = 0.010, *R. visakhapatnamense*; *p* = 0.007). On the other hand, there were no statistically significant differences under nitrogen-limited conditions (*R. sulfidophilum*; *p* = 0.053, *R. euryhalinum*; *p* = 0.253, *R. imhoffii*; *p* = 0.203, *R. visakhapatnamense*; *p* = 0.172). These results indicate that the PHA content per dry cell weight (wt%) did not increase during the incubation period under nitrogen-limited conditions, whereas a slight increase was observed for all the strains under nutrient-rich conditions. *R. sulfidophilum* showed higher PHA production after 5 days of incubation compared to other strains. We previously reported PHA production after 7 days of incubation using 9 purple non-sulfur bacteria strains (Higuchi-Takeuchi et al., 2016) and *R. visakhapatnamense* showed the highest PHA production and *R. sulfidophilum* exhibited the second highest PHA production. As shown in Figure S1, PHA production of *R. sulfidophilum* decreased after 6 days, whereas PHA production of *R. visakhapatnamense* increased gradually, leading to high PHA production of *R. sulfidophilum* after 5 days of incubation.

**Figure 1:**
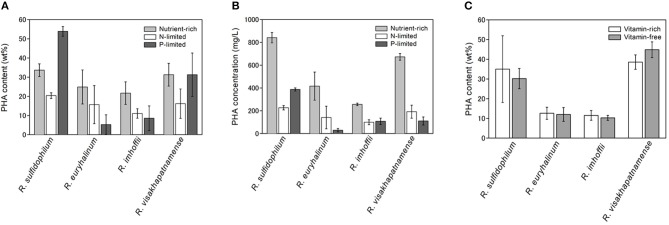
PHA production under nutrient limited conditions. **(A)** PHA content (wt%) and **(B)** PHA concentrations (mg/L) under nutrient-rich and nutrient-limited conditions. Four marine purple non-sulfur bacterial strains (*R. sulfidophilum, R. euryhalinum, R. imhoffii* and *R. visakhapatnamense*) were cultured in growth medium and then inoculated in nitrogen-limited (white bars) or phosphate-limited (dark gray bars) medium supplemented with 0.5% sodium acetate and in growth medium (light gray bars). **(C)** PHA content (wt%) under vitamin-free (light gray bars) and vitamin-rich (white bars) conditions. Data are the mean ± SD of at least three cultures.

The PHA content of the four strains was examined under phosphate-limited conditions (dark gray bars in Figure 1A). *R. sulfidophilum* exhibited higher PHA content (53.9 wt%) under phosphate-limited conditions than under nutrient-rich conditions. *R. visakhapatnamense* produced comparable PHA content (31.3 wt%) under phosphate-limited conditions and nutrient-rich conditions (31.3 wt%). These results suggest that PHA production is induced by phosphate limitation. However, all four strains grew poorly under phosphate-limited conditions because the extreme nutrient limitation caused poor cell growth and consequently reduced the total PHA production. As shown in Figure 1B, the PHA concentrations (mg/L of culture) were higher in the nutrient-rich medium than in the phosphate-limited medium.

It has been reported that *R. sulfidophilum* accumulates PHA under vitamin-free conditions (Chowdhury et al., 1996). Next, we examined bacterial growth in the absence of vitamins. Vitamin B12 and yeast extract, which contains high levels of B vitamins, were removed from the growth medium, and four strains were inoculated at an initial OD_660_ of 0.1 in the vitamin-free medium (lacking vitamin B12 and yeast extract). The PHA content could not be determined under these conditions due to poor growth of the marine purple non-sulfur bacteria. Therefore, we used a two-stage culture method for PHA production. In this method, PHA production was induced with improved productivity after sufficient cell growth was achieved. Bacterial cells were cultured at a starting OD_660_ of 1.0 in vitamin-free or vitamin-sufficient medium for 2 days, and the PHA content was measured. The PHA content of all four strains was not significantly different between vitamin-free and vitamin-sufficient conditions (Figure 1C). These results indicate that PHA production was not enhanced by vitamin-free conditions in the marine purple non-sulfur bacteria.

### Growth Dependent PHA Production

**Figure 2:**
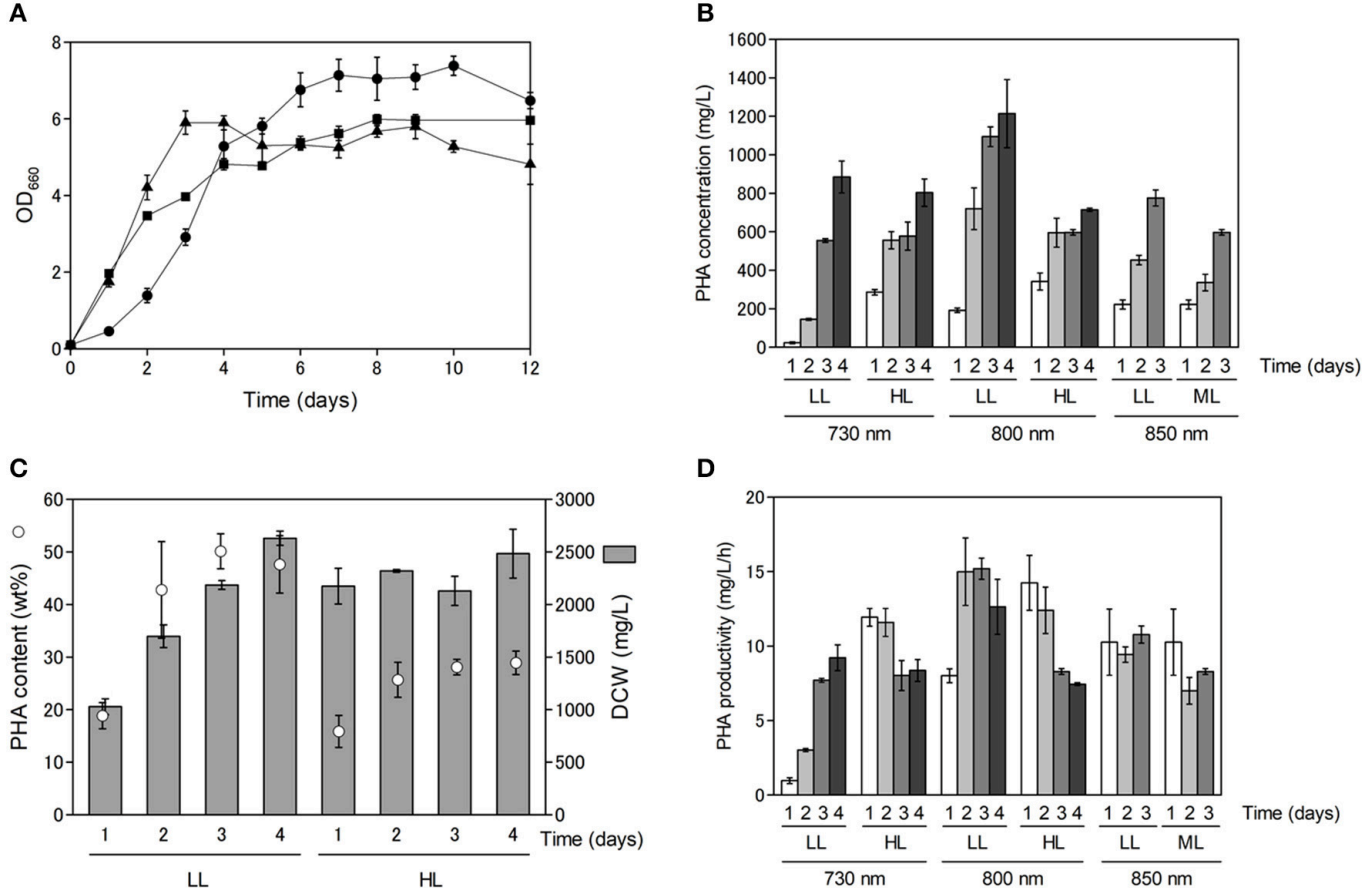
Cell growth and PHA production under different light conditions. **(A)** Growth of marine purple non-sulfur bacteria under different light conditions. Cells were inoculated in growth medium to an OD_660_ of 0.1 and cultured for 1 to 12 days under 8 W/m^2^ of 730-nm (black circles), 800-nm (black triangles) and 850-nm (black squares) LEDs. Data are the mean ± SD of at least three cultures. **(B)** PHA concentrations (mg/L) under different light conditions. Cells were cultured under low-light (LL: 8 W/m^2^), intermediate-light (ML: 20 W/m^2^, 850 nm) and high-light (HL: 50 W/m^2^) conditions. PHA concentrations were determined after 1 to 4 days of incubation. Data are the mean ± SD of at least three cultures. **(C)** Comparison between PHA content and DCW under 800-nm LED lighting. PHA content under low and high intensities of 800-nm LED light were plotted against DCWs. PHA contents and DCWs were determined after 1 to 4 days of incubation. **(D)** Time-course of PHA productivity (mg/L/h) under different light conditions. PHA productivities were determined after 1 to 4 days of incubation.

As shown in Figure S1, PHA production appeared to be dependent on cell growth. Purple non-sulfur bacteria use far-red light energy via bacteriochlorophyll absorption for growth, and hence, we examined the effect of light quality and light intensity on cell growth and PHA production. Cell growth and PHA production were examined using far-red LEDs at three different wavelengths (730, 800 and 850 nm) with *R. sulfidophilum* as a representative strain of marine purple non-sulfur bacteria. As shown in Figure 2A, the *R. sulfidophilum* cells grew well for up to 4 days of cultivation under illumination with LEDs at 800 nm (black triangles) and 850 nm (black squares), in contrast to growth under a 730-nm LED (black circles). This observation is plausible, because the absorption spectra of *R. sulfidophilum* exhibited peaks at 800 and 850 nm (Masuda et al., 1999), whereas 730-nm LED lighting was the best for bacterial growth after 5 days of cultivation. Generally, the penetration of light into photosynthetic microorganisms is limited at high cell densities because of the mutual shading of cells, and short-wavelength light can penetrate deeper than long-wavelength light. It is reported that short wavelengths of light (600–780 nm) reached the deeper part of the bioreactor, and energy of long wavelength light (800–850 nm) was lost in *R. sphaeroides* (Nakada et al., 1995). These results imply that short-wavelength LED lights (730 nm) might be able to penetrate the cells even at high cell densities.

Next, we examined the effects of light intensity on PHA production using LEDs of three different wavelengths in *R. sulfidophilum* (Figure 2B). Under all the conditions, the PHA concentrations (mg/L) increased with increasing incubation time. The PHA concentrations were highest after 4 days of cultivation (1.2 g/L) under low-light conditions with 800-nm LED lighting. The PHA content (wt%) was compared to dry cell weight (DCW) (mg/L) under low-light and high-light conditions with 800-nm LED lighting (Figure 2C). The DCWs were lower (or comparable) under low-light conditions than under high-light conditions. *R. sulfidophilum* exhibited higher PHA content (17-50 wt%) under low-light conditions than under high-light conditions (15-30 wt%), leading to the high PHA concentrations under low-light conditions shown in Figure 2B. The PHA content under low-light conditions with the three LED lights was plotted against the DCWs (Figure S2A). A linear correlation between PHA content and DCW was observed under low-light conditions (*R*^2^ = 0.6289), indicating that PHA production is dependent on cell growth under low-light conditions. On the other hand, the correlation coefficient between PHA content and DCW under high-light conditions was quite low (*R*^2^ = 0.0028, Figure S2B), indicating that there was no linear relationship. As shown in Figure 2C, the DCWs did not increase under high-light conditions during cultivation. Low PHA production under high-light conditions might be caused by poor cell growth.

PHA-producing bacteria can be divided into two groups based on culture conditions (Lee, 1996b). The first group requires nutrient limitation for PHA production. *Cupriavidus necator*, which is the most widely studied PHA producer, and many other bacteria belong to this group. The second group of bacteria does not require nutrient limitation and can accumulate PHA during growth. Some species, including *Alcaligenes latus*, mutant strains of *Azotobacter vinelandii* (Bormann et al., 1998) and recombinant *E. coli* strains (Slater et al., 1988), belong to the second group. The results of the present study indicate that marine purple non-sulfur bacteria belong to the second group, which do not require nutrient limitation for PHA accumulation. In the second group of bacteria, high PHA production at high cell densities leads to high PHA concentrations. As shown in Figure 2C, both PHA content and DCW reached saturation over 4 days of cultivation. Therefore, cultivation time is important for obtaining high PHA concentrations as well as for cell growth. The PHA productivity (mg/L/h) was calculated under all the conditions to determine the optimal incubation time (Figure 2D). The PHA productivity was highest after 3 days of cultivation under low-light conditions with 800-nm LED lighting. The relationship between PHA productivity and time of cultivation was analyzed under different light intensities. We found a negative correlation (*R*^2^ = 0.523) under high light conditions although no relationship was observed under low light conditions (*R*^2^ = 0.180). These results suggest that prolonged cultivation resulted in decreased productivity, although low-light conditions with 730-nm LED lighting seem to be the exception. Further optimization of light intensity and cultivation time is required to attain increased PHA production in marine purple non-sulfur bacteria.

### PHA Production Under Aerobic Conditions

**Figure 3:**
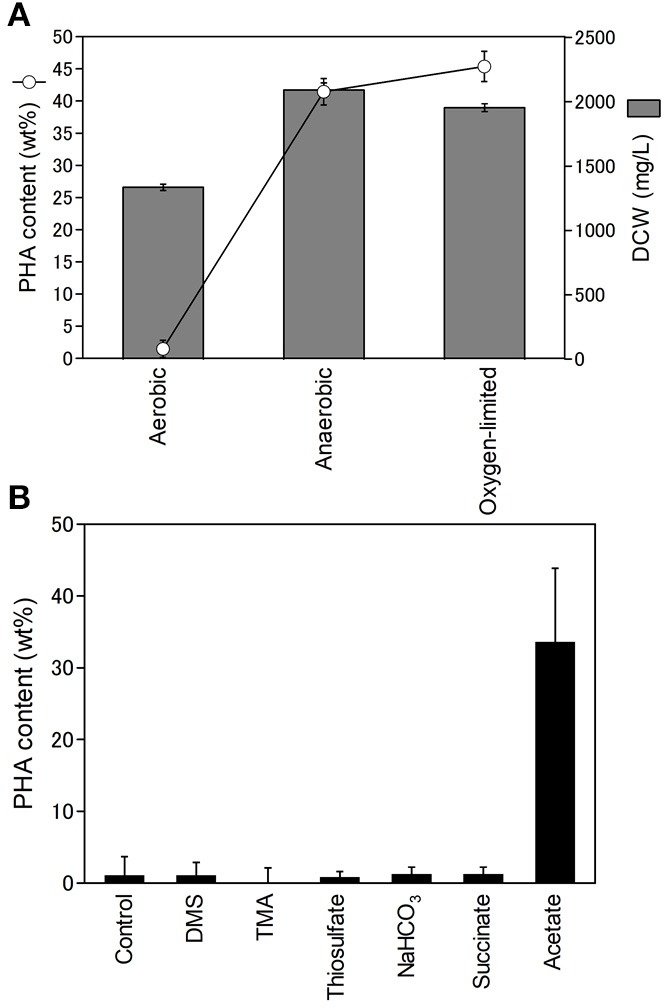
Effect of oxygen concentration on cell growth and PHA production. **(A)** PHA content (open circles) and DCW (gray bars) under different oxygen conditions. Cells were cultured under aerobic, anaerobic and oxygen-limited conditions. **(B)** Effects of reductants and carbon sources on PHA production under aerobic conditions. Cells were cultured under aerobic conditions in the presence of 0.3% malate and 0.3% pyruvate supplemented with 10 mM DMA (dimethylamine), 10 mM TMA (trimethylamine) and 10 mM thiosulfate as reductants and 20 mM sodium bicarbonate, 0.5% sodium succinate and 0.5% sodium acetate as carbon sources. Data are the mean ± SD of at least three cultures.

Purple non-sulfur bacteria are known to be able to grow heterotrophically in the dark under aerobic conditions. Next, we examined the effect of oxygen on PHA production. The PHA content and DCW of *R. sulfidophilum* were determined using cells grown under aerobic conditions in the dark, anaerobic conditions and oxygen-limited conditions in the light (Figure 3A). The DCW was highest under anaerobic conditions and lowest under aerobic conditions. We found that *R. sulfidophilum* cells accumulated very little PHA under aerobic conditions. *R. imhoffii* and *R. visakhapatnamense* also produced only 0.4 wt% and 1.8 wt% PHA, respectively, when cultured under aerobic conditions.

PHA is considered to be a store of reducing equivalents in addition to carbon. Therefore, the effects of additional reductants (DMS (dimethyl sulfide), TMA (trimethylamine), and thiosulfate) on PHA production were investigated under aerobic conditions. The addition of the three reductants did not affect PHA production under aerobic conditions (Figure 3B), suggesting that reducing equivalents are not key factors affecting PHA production under aerobic conditions. In the growth medium, malate and pyruvate were used as carbon sources. PHA production under aerobic conditions was also evaluated by the addition of three carbon sources (sodium bicarbonate, sodium succinate, and sodium acetate). The addition of sodium bicarbonate and succinate did not affect PHA production. Interestingly, *R. sulfidophilum* produced 33% PHA upon the addition of acetate under aerobic conditions (Figure 3B). These results indicate that the acetate-dependent pathway is important for PHA production under aerobic conditions. PHA production in the presence of reductants or carbon sources was also examined under anaerobic conditions (Figure S3). There were no noticeable changes upon the addition of TMA, DMS, and acetate under anaerobic conditions, suggesting that reducing equivalents and the acetate-dependent pathway are not involved in PHA production under anaerobic conditions.

### Expression Levels of TCA Cycle Enzymes

**Figure 4:**
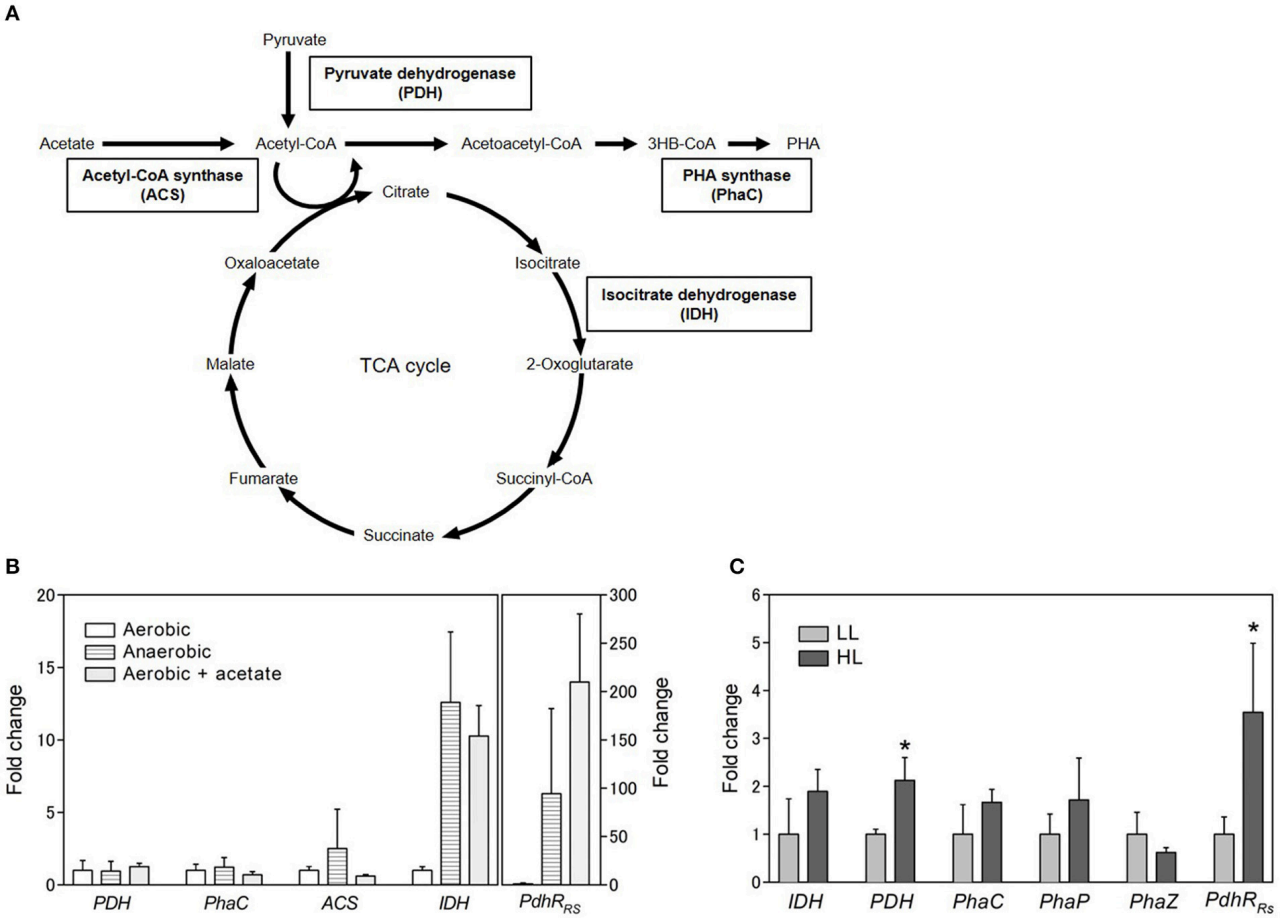
Gene expression analysis. **(A)** Schematic representation of carbon metabolic pathway. **(B)** Gene expression analysis of selected genes under aerobic conditions with or without acetate and anaerobic conditions. The expression levels are shown as the relative values compared with those of cells grown aerobically. The *rpoD* gene was used as a housekeeping gene to normalize the expression levels of target genes. **(C)** Gene expression analysis under different light conditions. Cells were cultured in low (light gray bars; 8 W/m^2^: 800 nm) and high (dark gray bars; 50 W/m^2^: 800 nm) light for 3 days. Data are the mean ± SD from at least three cultures. *P < 0.05.

To identify the gene involved in PHA production under aerobic and anaerobic conditions, gene expression levels were analyzed by quantitative RT-PCR. *PhaC* (PHA synthase), *PDH* (pyruvate dehydrogenase), *ACS* (acetyl CoA synthase), and *IDH* (isocitrate dehydrogenase) were selected for gene expression analysis (Figure 4A). PhaC is a key enzyme of the PHA biosynthesis pathway and polymerizes the PHA monomer. PDH catalyzes the conversion of pyruvate to acetyl CoA. ACS catalyzes the ligation of acetate and CoA to form acetyl CoA. The expression levels of *PDH, phaC*, and *ACS* were not significantly different between aerobic and anaerobic conditions (Figure 4B). Intriguingly, the expression level of *IDH* was lower under aerobic conditions than under anaerobic conditions. IDH catalyzes the oxidative decarboxylation of isocitrate to β-ketoglutarate and is an important enzyme in the TCA cycle. These results suggest that the TCA cycle activity decreased under aerobic conditions. Low PHA production under aerobic conditions might be caused by low activity of the TCA cycle. Low PHA accumulation in the presence of malate and succinate under aerobic conditions (Figure 3B) is plausible because these metabolites are intermediates of the TCA cycle.

### Effect of Acetate Addition

We also evaluated the effect of the addition of acetate on *IDH* expression. The *IDH* expression level increased 10-fold in the presence of acetate under aerobic conditions (Figure 4B). This finding suggests that the TCA cycle activity was enhanced by the addition of acetate under aerobic conditions. Recently, proteome analysis was carried out to investigate the metabolism of acetate under aerobic conditions in *R*. *rubrum* (Narancic et al., 2016). The results of the present study are consistent with the finding that the expression of succinate dehydrogenase in the TCA cycle was slightly increased by the addition of acetate. The TCA cycle is known to be regulated at two levels: the conversion of pyruvate into acetyl CoA, which is catalyzed by PDH, and the entry of acetyl CoA into the TCA cycle, which is catalyzed by citrate synthase. Acetyl CoA can be formed directly from acetate without the regulation of the TCA cycle when exogeneous acetate is supplied to the cells. PHA accumulation and enhancement of the TCA cycle by the addition of acetate might be explained by the direct production of acetyl CoA from acetate. Alternatively, the ethylmalonyl-CoA (EMC) pathway has been characterized as an acetate assimilation pathway in *R. sphaeroides* (Erb et al., 2007). In the EMC pathway, acetate is transformed to the TCA cycle intermediates L-malate and succinyl-CoA. The beta-ketothiolase (PhaA) and acetoacetyl-CoA reductase (PhaB) in the PHA biosynthesis pathway are shared with the EMC pathway. Therefore, induction of the EMC pathway by acetate would lead to the enhancement of PHA biosynthesis and the TCA cycle. The crotonyl-CoA carboxylase/reductase in the EMC pathway was identified in the genome of *R. sulfidophilum*. The EMC pathway activity might be enhanced by the addition of acetate in *R. sulfidophilum*, leading to PHA accumulation via the PhaA- and PhaB-catalyzed reactions.

IDH is regulated by the NADH/NAD^+^ and ATP/ADP ratios (Chen and Plaut, 1963). PDH is also known to be regulated by the NADH/NAD^+^ and ATP/ADP ratios and acetyl-CoA (Hansen and Henning, 1966; Shen and Atkinson, 1970; Luderitz and Klemme, 1977). Although the *PDH* mRNA levels did not change with the addition of acetate (Figure 4B), PDH protein expression was shown to be decreased by the addition of acetate in *R. rubrum* (Narancic et al., 2016). PDH regulation is reported to be controlled by PdhR (pyruvate dehydrogenase complex regulator) in some bacteria (Quail et al., 1994). PdhR is a member of the GntR family of transcription factors. The GntR family is widely distributed in bacteria and shown to be involved in sensing cellular signals in response to carbon utilization (Hillerich and Westpheling, 2006). A search for homologous amino acid sequences against *R. sulfidophilum* was performed using PdhR from *E. coli*, and a PdhR homolog was found in *R. sulfidophilum* (PdhR_Rs_). PdhR_Rs_ is composed of 256 amino acid residues and is conserved in *R. rubrum* and *R. sphaeroides* (Figure S4). The expression levels of *PdhR*_*Rs*_ were determined under three conditions. *PdhR*_*Rs*_ expression was highly enhanced (210-fold) by the addition of acetate under aerobic conditions (Figure 4C), suggesting that *PdhR*_*Rs*_ might be involved in the acetate-dependent pathway. The function of PdhR_Rs_ and its role in PHA production are currently unknown. PdhR is proposed to function as a master regulator of genes involved in energy production (Ogasawara et al., 2007). PHA production and the TCA cycle might be regulated by PdhR_Rs_ in *R. sulfidophilum*.

### Expression Analysis at Different Light Intensities

Gene expression analysis was also carried out using *R*. *sulfidophilum* cells grown under low and high light intensities with 800-nm LED lighting (Figure 4C). The expression levels of *IDH* and *PhaC* were almost the same between the different light intensities. The gene expression levels of other PHA biosynthesis genes, namely, *PhaP* (PHA granule associated protein) and *PhaZ* (PHA depolymerase), were also approximately the same under the two different light intensities. These results suggest that low PHA accumulation under high-light conditions was not explained by changes in the TCA cycle and the PHA biosynthesis pathway. *PDH* expression under high-light conditions was only slightly higher compared to low-light conditions. A more than 3-fold increase in the expression level under high-light conditions was observed for *PdhR*_*Rs*_, suggesting that PdhR_Rs_ might be involved in PHA production under high-light conditions.

Under high-light conditions, purple non-sulfur bacteria must dissipate excess reducing power to protect cells from photooxidative damage. PHA is considered to serve as an electron sink because PhaB uses NADH as a cofactor. However, PHA production under high-light conditions was lower than that under low-light conditions, as shown in Figure 2. Therefore, another pathway that dissipates reducing power must be enhanced under high-light conditions. It has been reported that hydrogen production catalyzed by nitrogenase increased under high-light conditions in *Rhodopseudomonas palustris* (Muzziotti et al., 2016), and PHA synthesis is considered to compete with hydrogen production in terms of utilization of reducing power (DePhilippis et al., 1992). Another possible pathway is the Calvin cycle, which requires reducing power. RuBisCO, which is an important enzyme in the Calvin cycle, is proposed to control the redox potential in *R. rubrum*, and PHA synthesis affected RuBisCO activity (Narancic et al., 2016). Hydrogen production and/or CO_2_ fixation might be induced under high-light conditions to dissipate excess reducing power. Further investigation is needed to elucidate the mechanism.

## Conclusion

In this study, we characterized PHA production in marine purple non-sulfur bacteria under various culture growth conditions (nutrient limitations, oxygen levels and light intensities and qualities). Marine purple non-sulfur bacteria could accumulate PHA during growth, and nutrient limitation did not lead to high PHA concentrations (mg/L of culture) unlike the well-studied PHA-producing bacteria. Low PHA production under aerobic conditions was caused by low TCA cycle activity and PHA production was strongly induced by the addition of acetate. Gene expression analysis revealed that the GntR family transcription factor PdhR (pyruvate dehydrogenase complex regulator) might have a role in the changes in the metabolic state.

## Data Availability

The raw data supporting the conclusions of this manuscript will be made available by the authors, without undue reservation, to any qualified researcher.

## Author Contributions

KN conceptualized the study and planned various experiments. MH-T planned and performed various experiments. KN and MH-T wrote the manuscript.

### Conflict of Interest Statement

The authors declare that the research was conducted in the absence of any commercial or financial relationships that could be construed as a potential conflict of interest.
