# Supplementary material for: Acetate-Inducing Metabolic States Enhance Polyhydroxyalkanoate Production in Marine Purple Non-sulfur Bacteria Under Aerobic Conditions
Source: Front Bioeng Biotechnol. 2019 May 28;7:118. doi: 10.3389/fbioe.2019.00118 (PMC6546801; doi:10.3389/fbioe.2019.00118)
Supplement: Supplementary file 1 [file Data_Sheet_1.pdf]

Supplementary Material

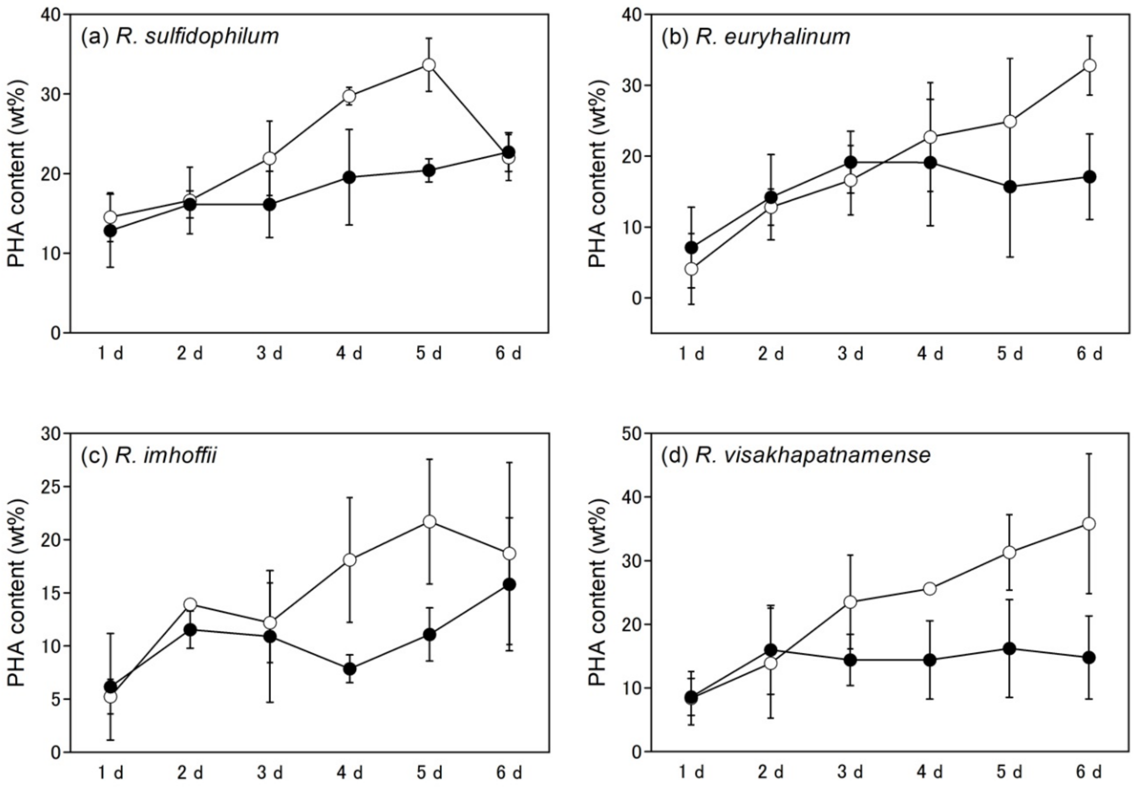

Supplementary Figure 1. Time course of PHA content (wt%) under nutrient rich (open circles) and nitrogen-limited conditions (closed circles). Four photosynthetic purple bacteria strains (*R. sulfidophilum*, *R. euryhalinum*, *R. imhoffii* and *R. visakhapatnamense*) were cultured in growth medium and then inoculated to growth medium or nitrogen-limited media supplemented with 0.5% sodium acetate for 1 to 6 days. Data are the mean ± SD from at least three cultures.

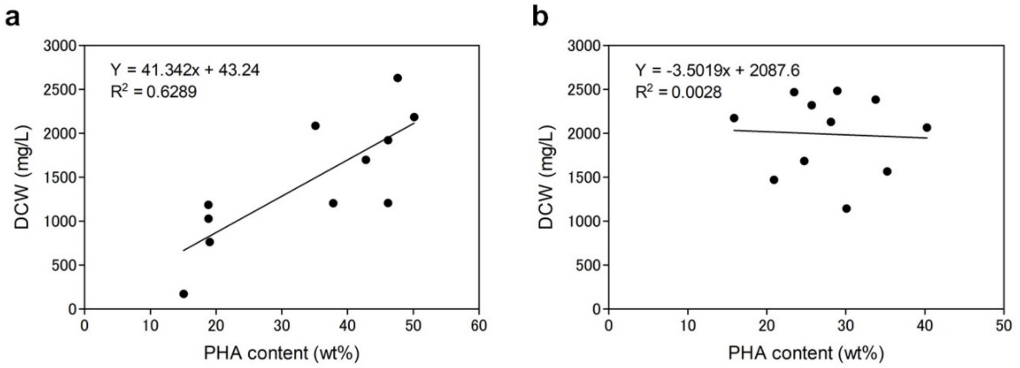

Supplementary Figure 2. Comparison between PHA contents (wt%) and DCW (mg/L) under low light (8 W/m^2) grown cells (a) and high light (50 W/m^2) grown cells (b). The regression lines and R^2 values were shown in each Figure.

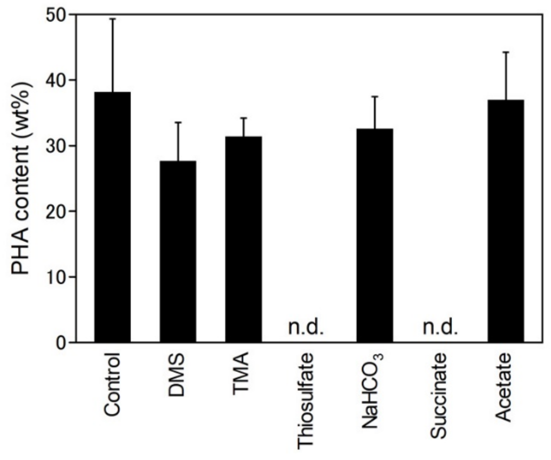

Supplementary Figure 3. Effect of reductants and carbon sources on PHA production under anaerobic condition. Cells were cultured under anaerobic conditions in the presence of 10 mM DMA (dimethylamine) and 10 mM TMA (trimethylamine) as reductants and 20 mM sodium biarbonate and 0.5% sodium acetate as carbon sources. n.d. = not determined. Data are the mean ± SD from at least three cultures.

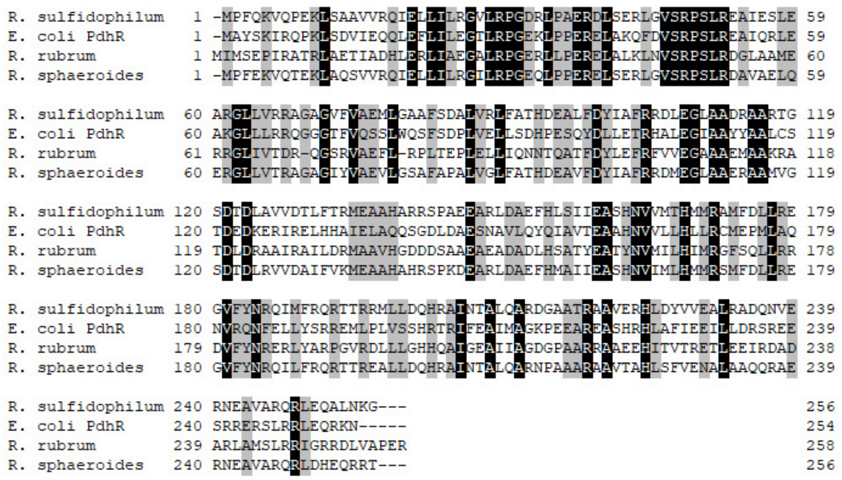

Supplementary Figure 4. Amino acid sequence alignment of PdhR homologs from *R. sulfidophilum*, *E. coli*, *R. rubrum* and *R. sphaeroides*. The amino acid sequences were aligned using the MUSCLE algorithm. Identical and similar amino acids are shown in black and gray boxes, respectively.

Table S1. Purple non-sulfur photosynthetic bacteria used in this study.

Resource No.	Organism
DSM4868	*Rhodovulum euryhalinum* (*R. euryhalinum*)
JCM13589	*Rhodovulum imhoffii* (*R. imhoffii*)
ATCC35886	*Rhodovulum sulfidophilum* (*R. sulfidophilum*)
JCM13531	*Rhodovulum visakhapatnamense* (*R. visakhapatnamense*)

Table S2. Primer sets used in this study.

Gene name	Primer A (5' – 3')	Primer B (5' – 3')	Product size (bp)
RpoD	CTTGTCCTCGATGAAATCG	GTCCGCAAGGTGATGAAGAT	108
IDH	ATCGACCAAGAACACCATCC	TCCTCGAATTCCTGCTCGTAG	64
PDH	TGCACATGTTCTCGAAGGAG	CGGCCATTGTCCTTGTACTTG	93
ACSA3	TGGGTCGAGAAATACGACCTG	TCCTCGAATTCCTGCTCGTAG	103
PhaC	ATTGAGCCCGTCGATATCCT	GCAGACCCATCCCTATTTCA	93
PhaP	TCGACGATCTTAACGTCCCT	CAATACGGAGACCAGCGATT	80
PhaZ	TGCGACGTCTATATCACCGA	CCGAGATGCTTGAGGAAATC	112
$PDHR_{RS}$	GCCGAATTCCATCTCTCGAT	CATGATCTGCCGGTTGTAGA	113
